# Importance of fasting blood glucose goals in the management of type 2 diabetes mellitus: a review of the literature and a critical appraisal

**DOI:** 10.15406/jdmdc.2018.05.00148

**Published:** 2018-07-20

**Authors:** Chandler J Tayek, Lavanya Cherukuri, Sajad Hamal, John A Tayek

**Affiliations:** Department of Internal Medicine, Los Angeles Bio-Medical Research Institute, USA

## Abstract

Prandial insulin has been essential for the improved management of the type 1 diabetic patient. Interestingly, many studies have evaluated the addition of prandial insulin to the type 2 diabetic patients with improved control. The greatest drop in A1c with the use of various type of prandial insulins have resulted in the decrease of 1.3% in the A1c measurement. Interestingly, none of the published trials with goal of fasting blood glucose (FBG) have ever obtained the goal A1c. Since a drop in FBG of 28.7mg/dl is equal to a 1% drop in A1c, a simple approach to obtain a target A1c would be to focus on the FBG (per ADA: Average Blood Glucose = A1c (%) x 28.7 - 46.7mg/d). However, average blood glucose requires multiple measurements and may be less accurate then using just a FBG. Since prandial insulin clinical trials have only demonstrated a drop in A1c by 0.3-1.3% the use of only a FBG to help patients get to goal may be easier to teach and to obtain. It might save time and money. Our hypothesis is that if patient obtain a FBG <100 mg/dl for 2-3 months then 70% will be at an A1c goal <7.0%. After a few months of good fasting glucose control the provider can use this equation (FBG+80)/30 to estimate A1c. For example, a FBG of 130mg/dl would be (130 + 80)/30 = 7.0%; or a FBG of 190 would be (190+80)/30 =eA1c 9% (estimate of A1c). While type 1 diabetes has a very complex daily glucose pattern, the approach to type 2 diabetics on insulin could become simplified.

## Introduction

Nearly all of the reviewed randomized clinical trials (24 of 25) have demonstrated that, the % A1c drops below 7.9%^1–25^ with the use of basal insulin. Prandial insulin’s have been used to treat type 1 diabetics and have become a common treatment for type 2 diabetics who are not at goal. Type 2 diabetic patients are in a prandial state for few hours with each meal. In comparison, the type 1 diabetic patients have larger and longer durations of glucose excursions after a meal which can account for 50% of the A1c value. The exact contribution of prandial glucose excursion in type 2 diabetic patients may be altering glucose levels to a smaller role base on the benefits of basal insulin suppressing hepatic and renal glucose production, dietary changes that have many patients eating less carbohydrate and the easy of administering basal insulin as a single once daily injection. For example, basal insulin administration has demonstrated that 68% of patients can get to an A1c of 7.0% or lower after 6 months of basal treatment.^2^ With the recent change in the A1c goal to be that of < 8.0%, the percentage of type 2 diabetic patient getting to goal may be approaching 90% with basal insulin alone. Data from the 30 clinical trials were reviewed for the percentage of patients with an A1c goal of ≤ 7.0%. Our primary hypothesis is that if providers obtain an average FBG of 90mg/dl, then approximately 70% will be at an ADAA1c goal ≤7.0%. Based on the newer ACP goal of an A1c < 8.0%, then approximately 90% will be at goal with if the FBG of 90mg/dl is obtained.

## Methods

Pubmed was searched as of June 2018 for clinical trials that have tested basal insulin analogue regimen in previously insulin naïve patients to evaluate the physiological effects and clinical outcomes using prandial vs basal alone insulin. Twenty five clinical trials using basal insulin were included in the final analysis. Twenty four of these clinical trials looked single basal insulin injection versus mixed dual insulin multiple injections in type 2 diabetic patients to identify if one regimen obtains a greater reduction in A1c values. All papers were included if the authors reported the percentage of diabetics at or below 7.0%^2–18,21–25^ or if this data was extractable from the published data.^19,20^ None of the trials reported % of patients at ACP goal A1c of < 8.0% but data was abstracted from the reported data.^4^

All the data reported percentage of patients at target A1c goal. Data was presented as mean± SEM. Significance was determined by ANOVA with significance defined as a p < 0.05. Linear regression was done by least squares and best equation determined that would permit FBG to predict % of patients at A1c goal. The regression equation demonstrated the relationship between the obtain FBG in each arm of the clinical trials to the percentage of patients at the ADAA1c goal of ≤ 7.0%. Interpretation of the relationship between FBG and the percentage at goal will developed and estimates of those at the new ACP goal of 8% was obtained (21).

## Results

Data was extracted from 26 clinical trials or reviews and entered into an Excel file for evaluation of the relationship between FBG and % of diabetics at goal as defined as by ADA and AACE of an A1c <7.0% and by ACP of an A1c < 8.0%. The majority of these trials were funded by manufactures of prandial insulin and the authors goal were to evaluate the benefit of adding prandial insulin to basal insulin to improve diabetic control. Eight clinical trials evaluate the addition of prandial insulin to basal insulin alone.^2–6^ The improvement in A1c ranged from 0.1 to 0.8%, and a larger proportion of patients obtained a goal A1c. However, there was a greater significant increase in the rate of hypoglycemia in most trials. In these eight clinical trial, the use of multiple injections of insulin resulted in a mean improvement in A1c of 0.36±0.21% (mean± SD, NS). While any improvement in A1c is likely better care for the patient, this may be obtained with a more focused approach of obtaining a low FBG goal now with the use of basal insulin and may reduce the incidence of hypoglycemia. This investigator believes that there may be an alternative explanation as described below:

Most trials were designed by companies funding rapid acting insulin with a goal to show superior benefits with rapid onset insulin as compared to standards of medical care. This interest could have produced a bias based on the fact that most studies increased rapid insulin treatment based on A1c measurements and not or fasting and post-prandial blood glucose concentration. For example, FBG goals were set to be less than 140, 126 or 120mg/dl respectively in 4 of the 8 trials.^4–7^ Since a change of 1% in the A1c measurement represents 28.7mg/dl change in the average blood glucose, a drop in FBG of 28.7mg/dl should represent a 1% drop in A1c (See explanation below). Having a goal of <120, <126 or < 144 respectively in 4 of the trials resulted in the highest FBG, (128mg/dl, 126mg/dl, 124mg/dl, 128mg/dl). (It is interesting that the goal for FBG was never achieved in these trials). If patients were able to obtain a FBG from 128mg/dl down to FBG 100mg/dl, then the FBG would have dropped 28mg/dl which would have resulted in an additional drop in A1c by approximately 1% (A1c = 28.7mg/dl):

- In four trials that set the strict goal of a FBG < 110 or lower, none of the trials obtained the goal FBG (114, 117, 122mg/dl, not-reported) respectively. Again, based on the fact that an A1c represent an average blood glucose (or fasting blood glucose) change of 28.7mg/dl, obtaining a more strict goal of 90mg/dl would have reduced the A1c by 1.0 additional percent.
- Most importantly, when FBG is reduced below 100 mg/dl, the patient’s endogenous first phase insulin response improves in most type 2 diabetic patients given intravenous glucose.^11^ Therefore; the diabetic patient may be able to better respond to acute glucose increases with endogenous insulin secretion. Since the majority of insulin secretion is trapped in the liver, the return of endogenous insulin secretion will blunt the post-prandial glucose rise as the insulin can effectively suppress hepatic glucose production.
- Approximately 70% of the endogenous insulin release is trapped in the liver which exerts it effect to suppress endogenous glucose production. Interestingly, at a FBS of 110mg/dl the ability of the beta cell to make additional insulin in the diabetic is impaired when compared to a fasting blood glucose concentration under 110mg/dl. Unappreciated by many clinicians, endogenous insulin secretion is restricted when FBG is above 110mg/dl. This is one of the reasons why AACE recommends that FBG be below 110mg/dl (See AACE guidelines 2018) in contrast the ADA that says the FBG should be between 70 and 130. This latter range is approximately 2.1% A1c delta (60/28.7 = 2.1).
- In a study of 785 diabetic patients, basal insulin was given for 14-weeks before being randomized to three different rapid insulin arms. After this 14-week run-in period, 47% of the patients on basal insulin alone were not randomized to any of the rapid treatment arms because they were at A1c goal (≤ 7% of lower; Ref 12). Extrapolation of the percentage at goal with FBG 130 was approximately 90%.

**Figure 1:**
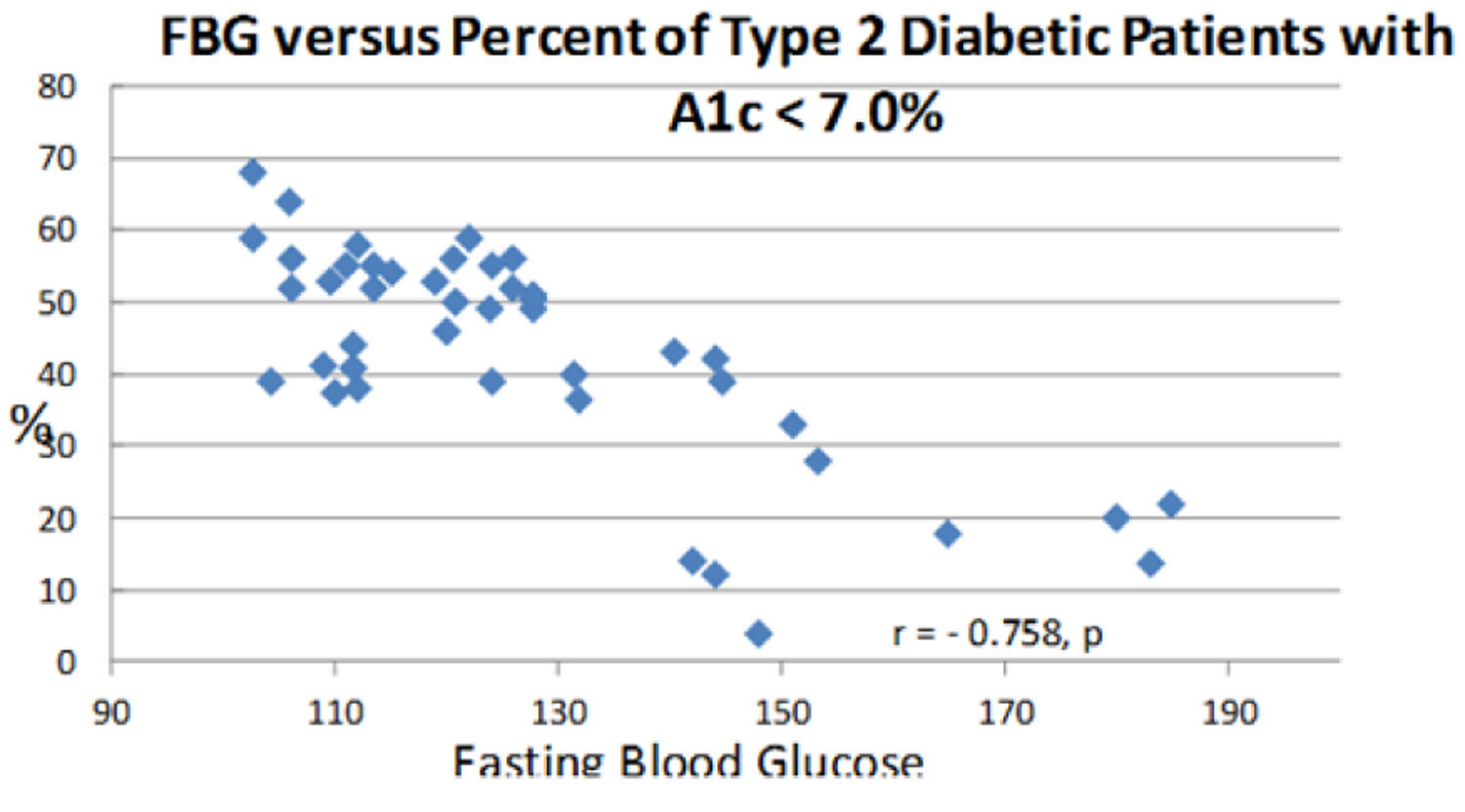
FGB versus Percent of type 2 diabetic patients with A1c<7.0%

In an attempt to validate the approach, the results from the 26 publications resulted in 54 data points including both FBG and % of patients with A1c ≤ 7.0%. Each data point was the mean for the treatment and the control arm of the study. The data demonstrates a significant regression correlation (Figure 1). Interestingly, the equation is simple to use: (174 – 1.07 x FBG = Percentage of population with A1c ≤ 7.0%). For example, FGB of 90mg/dl would be obtained and the formula would estimate that (174 – 1.07 × 90) 77.7% would be at goal A1c ≤ 7.0%. Based on Table 1, the 28mg/dl increase blood glucose would translate into an approximate A1c < 8.0% which is the new ACP guidelines for diabetic patients to be at goal. Therefore, approximately 90% of patients with a FBG of 120mg/dl should be at a goal A1c of 7.9% or lower.

## Discussion

The ADA goal for FBG concentrations are the same for patients with both type 1 and type 2 diabetes mellitus. The goal range is between 70 and 130mg/dl. This 60mg/dl range between 70-130mg/dl accounts for about a 2.1% change in A1c for most type 2 diabetics. For the type 1 diabetic, the wide range may not account for 2% in A1c control because the glucose variability is quite high for type 1 patients when compared to type 2 diabetic patient. While having a target FBG of 90mg/dl may be considered too tight a goal for the DM-1, but this may be a safe way to approach patient with DM-2. We know hypoglycemia is a common reason for ER visits. The majority of these visits are likely due to prandial insulin (when not eating) or sulfonylurea mediations. In comparison, both AACE and Canadian Medical Association have a FBG range more consistent with the physiological process of insulin secretion (as discussed below) between 110 and 90mg/dl.

Beta cell response to intravenous glucose is absent at a fasting blood glucose of 115mg/dl to 140mg/dl.^11^ At FBG of 100 to 114mg/dl, the insulin response to glucose is minimal. In comparison, at a FBG of 79 to 89mg/dl and at a FBG between 90 and 99mg/dl, the first phase insulin response is robust.^11^ First phase insulin response is essential to regulate hepatic glucose production rate which is responsible for FBG concentration. Adding adequate basal insulin suppressed hepatic glucose production rates to such a level as the FBG decreases.

First phase insulin response occurs when fasting glucose concentrations are below 111mg/dl. Unfortunately, none of the eight clinical trials obtained a FBG <110mg/dl. A FBG goal was set for all 26 clinical trials, none of the studies obtained the goal mean FBG. In an additional study similar to the Davidson study,^11^ if you treat with basal insulin (glargine) for 6 months with an average FBG of 120mg/dl, then 46% of your patients will have an A1c ≤ 7.0%.^14^ In an attempt to help refocus the importance of FBG and A1c, Table 1 list to goal FBG to obtain an A1c concentration. Instead of using the 28.7mg/dl per A1c, Table 1 has been simplified for every 30mg/dl is 1% A1c. (ADA table is similar for mean blood glucose if you add approximately 30 mg to Table 1). Obtaining a FBG of 120 for a 6-month period resulted in a mean A1c of 6.7% in a large clinical trial.^13^ This number is exactly what would be expected in type 2 diabetic patients where 18 hours of the 24-hour day reflects fasting state where fasting blood glucose accounts for 80% of the glucose area under the curve. In comparison, after 12 weeks of therapy the average fasting blood glucose of 148mg/dl resulted in an A1c of 8.6%.^14^ It is unclear why the A1c of 8.6% was higher than predicted (7.8%). The half-life of the RBC is 120 days so that at least one half-life (120 days or 17 weeks) would have been a better period of time to demonstrate benefit between fasting blood glucose and A1c (Table 1). Likewise, obtaining a FBG of 111mg/dl after 13 weeks resulted in an average A1c of 7.8% and not 6.3% as might be predicted in Table 1.^15^ In a third study, 14 weeks of glargine resulted in a FBG of 132mg/dl and an A1c of 8.0% where one might expect a 7.2% if the treatment period was at least 120 days.^12^ The failure of the A1c to reflect the FBG is due to the 120 half-life of the glycosylated RBC which can delay an accurate A1c measurement. Treating to target FBG takes time and most clinical trials did not measure the A1c after 120 days of being at a target FBG.

In addition to the Table 1, this can be translated into a simple equation where (FBG+80)/30= A1c; for example, a FBG of 130 would be (130+80)/30 = 7.0%; or a FBG of 190 would be (190+80)/30 =9%. In these three studies, A1c measurements would have likely resulted in an lower A1c of approximately 6.7 for the first study,^12^ 7.6% for the second study^14^ and 6.3% for the third study,^15^ and 7.2% for the Davidson Study,^12^ respectively. The fact is that the FBG in DM-2 reflects the current glycemic goal but the A1c reflects that last 120-day average (3-4 month average) which lags behind the current improvement in control. In an attempt to validate the argument that for type 2 diabetics, the FBG is the best measurement to obtain better control was based on the observation that none of the studies of type 2 diabetic patients have demonstrated more than a 1.3% point decrease in A1c with prandial coverage.^2–8^ In the most recent study, the addition of 3 shots a day of Glulysine, the delta A1c was −0.4% reduced.^12^ While more patients were at goal, the risk for hypoglycemia was increased. An alternative approach may be obtained by drop-in the FBG by only 12 points (138 down to 126mg/dl) with the use of additional basal insulin as the 12 point drop would drop the A1c by 0.4%. Likewise in a second study, the addition of a single injection of glulisine resulted in an improvement of the A1c by 0.26%.^15^ Unfortunately, data interpretation is difficult with regards to this basal bolus study since the authors only used 12 weeks to obtain a FBG of 148 (estimated A1c of 7.6%) but the authors reported an A1c of 8.8%. Based on fasting blood glucose concentration, the A1c would have been 7.6% if the authors maintained the basal injections for a sufficient period to allow the A1c to account for the 120 day half-life of A1c. In argument, approximately 2-3 half-lives would be required for one to be certain that the FBG is accurately reflected in A1c measurement. The addition of Aspart to basal insulin resulted in a drop of A1c to an average of 7.6%. This was a 1.2% drop in A1c and could be considered a marked improvement in control. However, with the 1.2% drop in A1c was accompanied by a multifold increase in hypoglycemia. This is not to mention the additional costs for the rapid acting insulin and potential ER visits for severe hypoglycemia. Recent data suggests that intensive versus standard blood glucose control in North American type 2 diabetic patients increased all-cause mortality (1.21, p <0.05), CV mortality (1.41, p <0.05) and an associated OR of 3.52, P <0.05) for severe hypoglycemia.^16^ Consistent with all the published clinical trial is the inability to obtain goal FBG. The push to fund research in prandial insulin from market forces and the inability of the A1c to reach a steady state may have over inflated the apparent effectiveness of bolus insulin added to a basal regiment. Using basal insulin alone and obtaining a FBG of 148mg/dl resulted after 12 weeks with only 4% of the population with an A1c concentration below 7%.^12^ After 13 weeks with a final FBG of 111mg/dl, 14% of the patients were below an A1c of 7%.^13^ After 14 weeks, with a final FBG of 132mg/dl, 36% of the patients had an A1c at or below 7.0%.^12^ Most importantly, after six months with a final fasting blood glucose of 120mg/dl, 46% of the patients had an A1c less than or equal to 7.0%.^13^

As shown in the regression equation, if a FBG of 90mg/dl is obtained the 78% of patients on basal insulin alone should be at goal. Therefore, if one gets the FBG to 90mg/dl within a 4-month period, then some 88% should be at goal. The average A1c after 6 months was 6.7% which is consistent with Table 1 estimate relationship between FBG and A1c. It is important to point out that approximately 17 weeks (4 months) is the minimal time (120 days) that is needed with a consistent FBG to see the effects of glycemia on A1c concentration. Asking the clinician to focus on FBG will simplify the goal for the patient, family and provider.

Emphasis has been placed on the prandial insulin which has resulted in a delay in the advancement of insulin management seen in general internal medicine and family medicine clinics. If you make a goal simple for your patients and aim for fasting blood glucose of 90mg/dl, 88% should obtain an A1c less than 7.0%. This can be done easily by a single morning injection of basal insulin. The fault in many of the bolus studies is the failure to have a “basal only arm” once they were randomized to rapid insulin regimen and also not obtaining the stated fasting blood glucose goal. Not to point blame on much of the published data, but if one wanted to promote prandial insulin treatment, one could accept FBG in the higher range to allow the prandial insulin to show a greater benefit (larger area under the glucose excursion curve). This would also prevent hypoglycemia since you are starting at a glucose level of 130-140 with prandial injections and meals. Unfortunately, just because a study was not done and goals obtained should not provide reassurance in the obtained outcomes, poorly designed data should not drive medical care. The use of rapid insulins without continuing a placebo arm,^12^ having a very short lead in time,^3–10,12–14^ all contribute to the false impression that prandial insulin treatment in the type 2 diabetic is important. In fact, Buse et al.,^3^ demonstrated that glargine treatment alone as compared to the use of BID (75/25 lispro protamine suspension/25% lispro insulin) resulted in similar A1c concentration with one less injection per day.^13^ These authors keep the FBG at 120mg/dl with glargine despite the fact that a small increase in glargine would have reduced the FBG to goal. Never-the-less, the additional drop in A1c based on the basal bolus insulin was 0.3%. This same drop could have been obtained if the authors had obtained the drop of fasting blood glucose by 10mg/dl (from 120-110mg/dl) as currently recommended by AACE guidelines. An entire 1% drop in A1c would have been seen if the goal FBG 0f 90mg/dl was obtained.

Only one study has targeted a FBG between 70-90mg/dl and obtained a FBG of 79mg/dl. After 20 weeks, 64% obtained an A1c <7%. In those who obtained a FBG between 70 and 110, 54% obtained an A1c < 7%.^17^ Average A1c was 6.7% in the 70-90mg/dl arm and 7.0% in the 70-110mg/dl arm. As in most other studies, the goal FBG was not obtained and the mean FBG at the end of the study were 106mg/dl and 111mg/dl. If the authors were to obtain a mean FBG goal of 80mg in the intense arm, then the A1c would have been approximately 0.9% better than reported. In addition, the short term 12-week period of a good FBG was not been long enough to complete the 120 day duration for A1c steady state. Figure 1 reflects the actual relationship between final FBG and A1c as well as the predicted A1c based on FBG obtained (if adequate time i.e. 120 days) had elapsed at that FBG (Figure 1). Lastly, recent studies have shown that small changes in FGB results in significant difference as earlier studies.^12–15,17–19,26^ Our hypothesis that 70% of the patients would be at ADA goal A1c ≤ 7.0% if the FBG of 90 was obtained is supported by the data in Figure 1. This figure is an underestimate of the real number at goal based on the fact that most studied did not sustain FBG for a 4 month period prior (RBC life span) to measurement of A1c level. Using the newer ACP guideline A1c goal < 8.0% then Figure 1 shows that some 90% can be at goal if the FBG is 120. This is consistent with the new ADA guidelines that basal insulin should used to obtain a target FBG for several months. After several months, and I would suggest 4 to 6 months, then measure the A1c to see if at A1c goal.

## Conclusion

Basal insulin is a safe and easy way to obtain goal A1c without the risk of severe hypoglycemia. A goal of FBG of 110 should be obtained initially for 2-months to avoid excessive beta cell recover and subsequent hypoglycemic. After 2-3 months the target FBG could be lowered to 90mg/dl with sufficient time elapsed to measure the A1c. ADA recommends measurement of A1c every 3 months when the patient is not at goal. It important to point out that the A1c takes 120 days (approximately 4 months) to get to a new steady state. Checking A1c every 3 months may be too soon to evaluate the effect of basal insulin if the FBG has not been at goal for 2-3 months. Using the simple formulates to estimate control may be helpful: (FBG+80)/30 to estimate A1c (See Table 1).

Patience is a virtue when treating patients with type 2 diabetes who are on insulin. Clinicians should focus of fasting glucose concentration with medical visits and not evaluate the A1c, as sufficient time may has not elapsed to see ones goal A1c. With the new recommendation by ACP to obtain an A1c between 7.0 to 7.9% the ability to obtain this should be easier as one can still target a FBG of 130 and get your patient to goal. ADA 2018). Simplifying the goal of treatment in diabetes to FBG provides the patient with more selfcontrol at obtaining this goal and gives them an easy home monitor to keep a check on their diabetes. The target of FBG in type 2 diabetics should improve compliance, reduced costs associated hypoglycemia, use of expensive prandial insulin and cost associated with frequent glucose monitoring at home.

## Figures and Tables

**Table 1:** Relationship between FBG and A1c

Fasting glucose (mg/dl)	A1 c (%)
100	6
130	7
160	8
190	9
220	10
250	11
280	12
310	13
340	14
370	15
400	16
430	17
460	18
490	19

## Abbreviations:

FBG: fasting blood glucose
ADA: average blood glucose
ACP: autologous conditioned plasma
A1C: hemoglobin A1c
AACE: American association of clinical endocrinologists
